# Knockdown of *CDKN1C (p57^kip2^)* and *PHLDA2* Results in Developmental Changes in Bovine Pre-implantation Embryos

**DOI:** 10.1371/journal.pone.0069490

**Published:** 2013-07-22

**Authors:** Ashley M. Driver, Wen Huang, Jenna Kropp, Francisco Peñagaricano, Hasan Khatib

**Affiliations:** 1 Department of Dairy Science, University of Wisconsin-Madison, Madison, Wisconsin, United States of America; 2 Department of Genetics, North Carolina State University, Raleigh, North Carolina, United States of America; 3 Department of Animal Sciences, University of Wisconsin-Madison, Madison, Wisconsin, United States of America; Justus-Liebig-Universität, Germany

## Abstract

Imprinted genes have been implicated in early embryonic, placental, and neonatal development and alterations in expression levels of these genes can lead to growth abnormalities and embryonic lethality. However, little is known about the functions of bovine imprinted genes during the pre-implantation period. Therefore, the objective of this study was to assess the influence of altered expression of imprinted genes on developmental progress of embryos using small interfering RNA (siRNA). Expression levels of 18 imprinted genes (*MAGEL2*, *UBE3A*, *IGF2R, NAP1L5, TSSC4, PEG3, NDN, CDKN1C, PHLDA2, MKRN3, USP29, NNAT, PEG10*, *RTL1, IGF2, H19*, *MIM1,* and *XIST*) were compared between embryos reaching the blastocyst stage and growth-arrested embryos (degenerates) using quantitative real-time PCR (qRT-PCR). Ten genes were found to be differentially expressed between blastocysts and degenerates. The *CDKN1C* gene showed the highest upregulation in blastocysts whereas *PHLDA2* was highly expressed in degenerates. To assess whether the observed differential gene expression was causative or resultant of embryo degeneration, these genes were selected for functional analysis using siRNA. Injection of siRNA specific to *PHLDA2* into one-cell zygotes resulted in a substantial increase in blastocyst development, whereas injection of *CDKN1C*-specific siRNA resulted in a 45% reduction (P = 0.0006) in blastocyst development. RNA-Seq analysis of *CDKN1C*-siRNA-injected vs. non-injected embryos revealed 51 differentially expressed genes with functions in apoptosis, lipid metabolism, differentiation, and cell cycle regulation. Gene ontology analysis revealed nine pathways related to cell signaling, metabolism, and nucleic acid processing. Overall, our results show that proper expression levels of the imprinted genes *CDKN1C* and *PHLDA2* are critical for embryo development, which suggests that these genes can be used as markers for normal blastocyst formation.

## Introduction

Although the use of in-vitro fertilization (IVF) has been steadily increasing for the past three decades**,** low success rates of early embryonic development and successful pregnancy have been reported for both bovine and human. Approximately 40% of fertilized bovine oocytes reach the blastocyst stage by day 8 of development and of these only 45% result in pregnancy [1]. As such, there is a need to better understand the mechanisms affecting proper embryo development. To identify genetic factors affecting fertilization success and embryo quality, our laboratory utilizes an established IVF system for genomic and transcriptomic profiling of bovine embryos [2]. Using this controlled *in vitro* system, we have discovered DNA variations and aberrant gene expression that are associated with fertilization success and embryonic development [2]–[7]. These findings provide valuable genetic and biological markers for fertility of dairy cattle. Nevertheless, the causal genetic variants and molecular mechanisms of differential gene expression are yet to be revealed.

In a previous study that used microarray expression analysis, we compared the transcriptomes of developed IVF blastocysts to degenerate embryos, which do not properly complete the transition from morula to blastocyst [3]. We found a number of genes and pathways that were altered in degenerate embryos, among which the imprinted gene *PHLDA2* (pleckstrin homology-like domain, family A, member 2) was significantly up-regulated in by more than eight-fold compared to blastocysts [3]. Imprinted genes are of particular interest due to their reported roles in embryonic, placental, and neonatal growth [8]. Evidence for the importance of proper imprinted gene function can be seen in animal model studies where disruption or knockouts of particular imprinted genes have resulted in abnormal progeny or lethality in utero [9], [10]. Imprinted genes have also been implicated in livestock development, as differential expression of these genes has been associated with aborted and abnormally developed bovine clone fetuses [11], [12]. However, there is limited information regarding the role of these genes during the early developmental period.

In this study, we report the association between altered expression of several imprinted genes and blastocyst formation as a measure of proper embryo development. In order to interpret whether expression levels were causative or resultant of embryo degeneration, the most differentially expressed genes were silenced through microinjection of small interfering RNA (siRNA), and embryo quality was recorded for injected and control embryos. The siRNA method utilizes the cellular machinery to either rid the cell of foreign double-stranded RNA or to cause translational repression via microRNAs [13]. In addition, RNAi machinery can be utilized to target specific mRNA sequences and cause degradation through introduction of double-stranded RNAs of 9–29 nucleotides [14]. Currently, there are very few studies that have used siRNA in bovine embryos and no available information regarding the function of imprinted genes during this developmental period [15]–[19]. Additionally, it is important to assess any global effects of single-gene knockdown. Thus, siRNA-injected embryos were compared to controls using RNA-sequencing to determine the pathways downstream of the silenced gene that may have been altered. The identification of these genes and pathways can provide a clearer picture of how an individual gene fits into the biological circuitry of the developing embryo.

## Results

### Association of Expression Levels of Imprinted Genes with Pre-implantation Bovine Embryo Development

**Figure 1:**
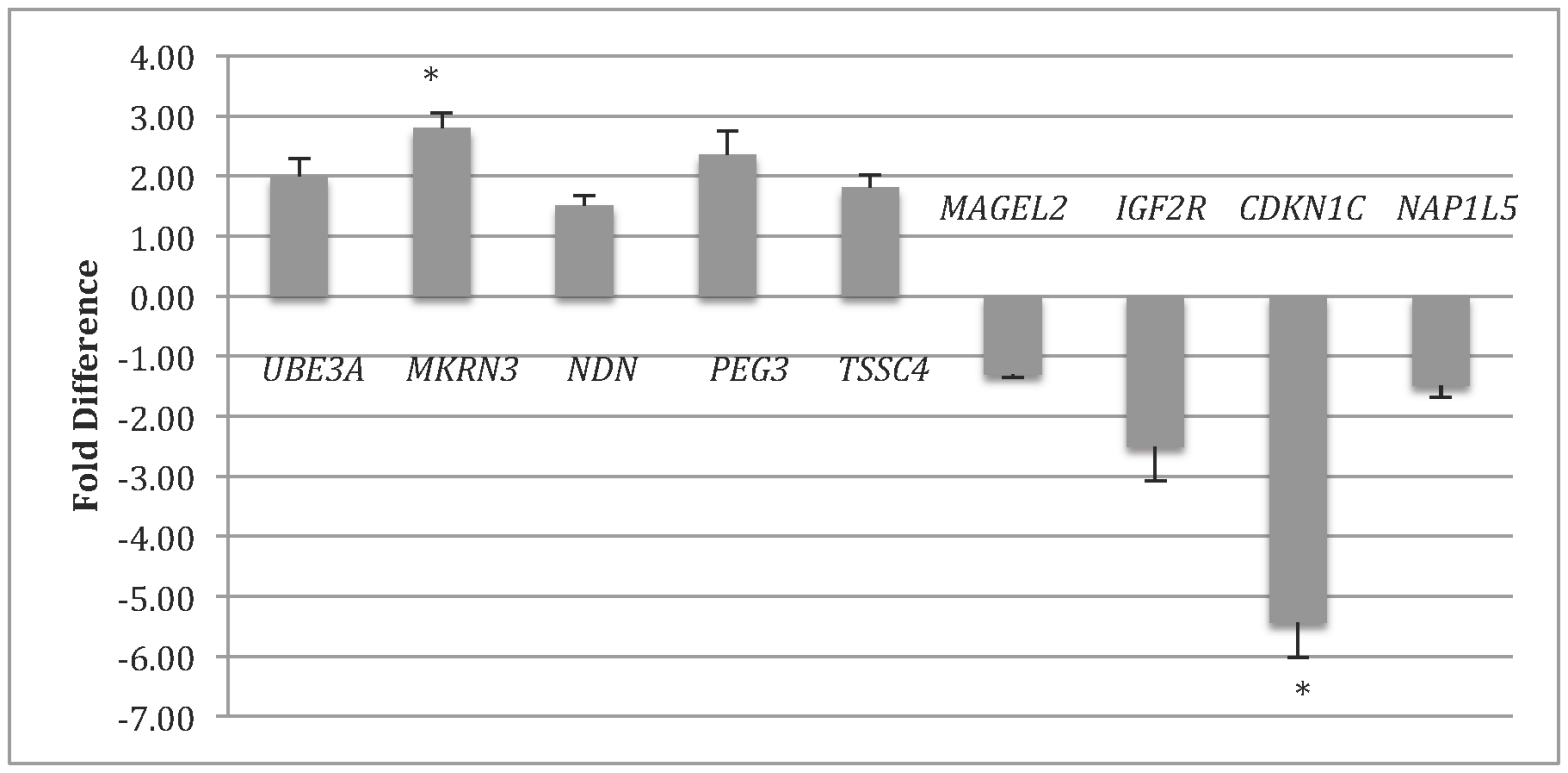
Mean+S.E.M. for fold difference of degenerative relative to blastocyst embryo pools. Each bar represents the values across four sets of embryo pools (n = 20 embryos per pool, two sires used). All samples were done in quadruplicates and normalized to *GAPDH* tested in the same cDNA samples. Expression calculations were done using the 2^−ΔΔCt^ method [60]. Bars above the ”0″ represent genes that were up-regulated in degenerative embryos while bars below the ”0″ indicate down-regulated genes. * P<0.05.

In a previous study, we used microarrays to profile gene expression of IVF embryos showing distinct developmental statuses. Among the differentially expressed genes, *PHLDA2* was found to be significantly up-regulated in degenerate embryos as compared to normally developed blastocysts in both microarray and qRT-PCR experiments [3]. Given that *PHLDA2* is imprinted and that imprinted genes have key roles in embryo development, we sought to assess whether other imprinted genes may show association with developmental status of the embryo. Nine genes (*MAGEL2*, *UBE3A*, *IGF2R, NAP1L5, TSSC4, PEG3, NDN, CDKN1C,* and *MKRN3*) were detected in pools of blastocyst and degenerate embryo populations with quantifiable differential expression. *NDN, TSSC4, UBE3A, PEG3*, and *MKRN3* were found to be up-regulated in degenerate embryos showing average 1.5±0.17-fold, 2.0±0.22-fold, 2.0±0.31-fold, 2.4±0.30-fold, and 2.8±0.26-fold differences between pools, respectively (Figure 1). The genes *MAGEL2, NAP1L5*, *IGF2R*, and *CDKN1C* showed average 1.3±0.04-fold, 1.5±0.2-fold, 2.5±0.57, and 5.4±0.58 -fold up-regulation in blastocysts, respectively (Figure 1). Of those differentially expressed, *MKRN3* (P = 0.031) and *CDKN1C* (P = 0.035) showed statistically significant differences in expression between blastocyst and degenerate embryos, and *PEG3* had differential expression that was close to a level of significance (P = 0.057). Four genes (*USP29, NNAT, PEG10*, and *RTL1*) had very low expression in embryos making it impossible to quantify differences accurately, while three genes (*IGF2, H19*, and *MIM1*) had undetectable levels of expression in our embryo populations. To reveal the mechanisms underlying the differential expression observed in pre-implantation embryos, *PHLDA2* and *CDKN1C* were selected for further functional analysis.

### DNA Methylation of *PHLDA2* is Associated with Differential Expression and Tissue Specificity

**Figure 2:**
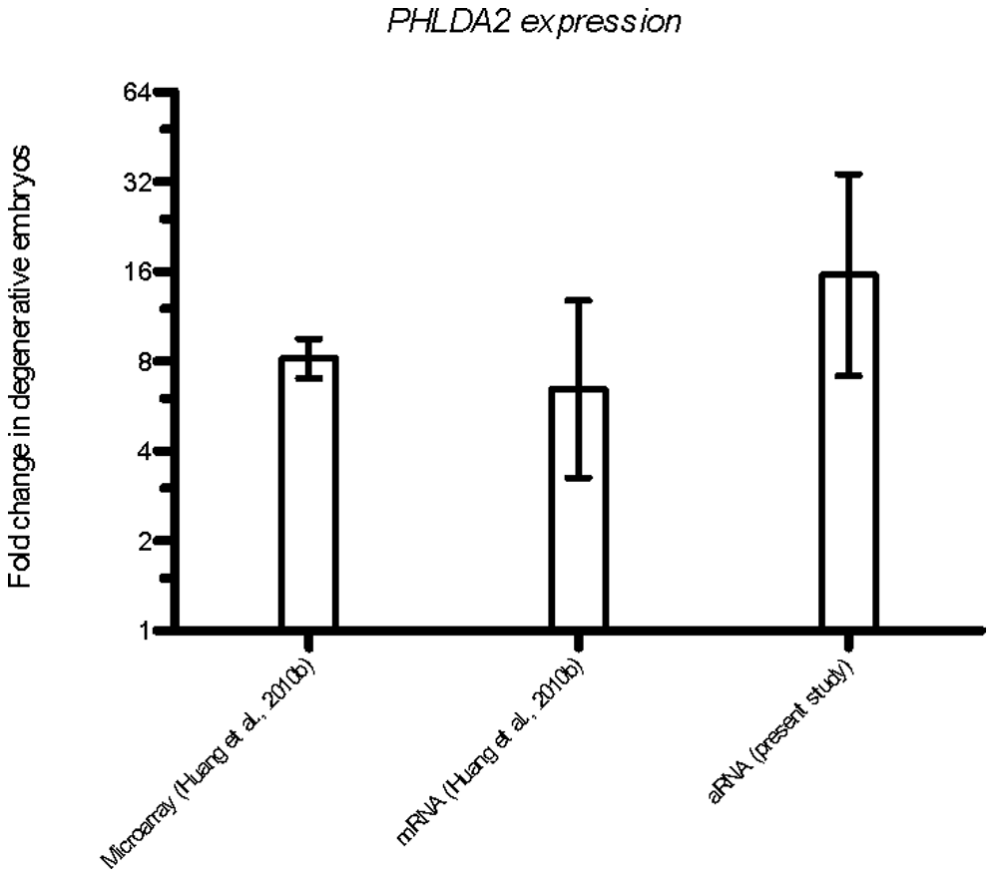
Differential expression of PHLDA2 in IVF blastocysts and degenerate embryos. Data for PHLDA2 expression from microarrays and mRNA was obtained from Huang et al. [5]. Expression of *PHLDA2* was normalized to *GAPDH* and shown as fold change (± SD) in degenerate embryos.

In this study, the up-regulation of *PHLDA2* was reconfirmed in three additional pairs of biological replicates, showing 15-fold higher expression in degenerates compared to blastocysts (Figure 2). Although the magnitudes of difference varied between pairs of embryos and between studies, *PHLDA2* expression was consistently higher in degenerate embryos than in blastocysts. These results clearly suggest an association between aberrant *PHLDA2* expression and abnormal early embryonic development in IVF embryos.

**Figure 3:**
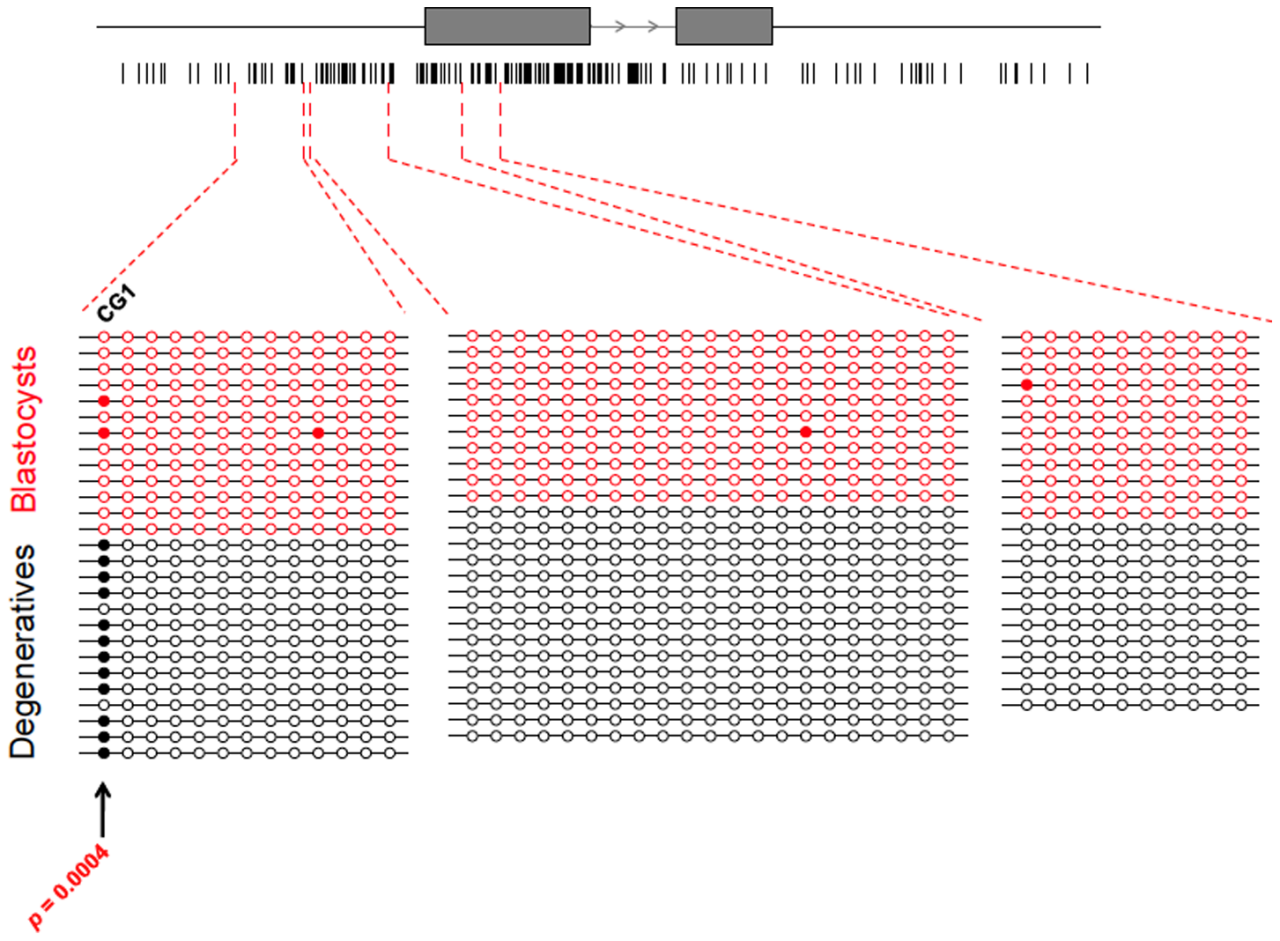
DNA methylation of *PHLDA2* in IVF embryos. Exons of *PHLDA2* are indicated by grey boxes and the direction of transcription is indicated by arrowheads. Vertical bars below the gene model represent CpG sites near and within *PHLDA2*. The CpG island overlapping with *PHLDA2* is shown as an open rectangular box. For methylation analysis, each line connecting circles represents a single clone. Filled circles indicate methylated CpGs while open circles represent unmethylated CpGs. Clones derived from blastocysts and degenerate embryos are shown in red and black respectively. CpG sites tested for differential methylation are marked by arrows. Significant differential methylation of CpG sites are indicated by p values.

Because of the importance of DNA methylation in regulating transcription–particularly that of imprinted genes–we measured the methylation of cytosines at CpG sites near or within the *PHLDA2* gene by bisulfite sequencing. A CpG island highly enriched for CpG sites was found to overlap with the first exon and first intron of *PHLDA2* as well as upstream of the start of the transcript. DNA methylation analysis of blastocysts and degenerate embryos revealed rare methylation of CpG cytosines (Figure 3). However, one CpG site upstream of the *PHLDA2* transcription start site was highly methylated in degenerate embryos relative to blastocysts (Figure 3, *P* = 0.0004). To test whether DNA methylation was associated with the tissue specificity of *PHLDA2* expression and imprinting, we measured methylation of DNA from heart, where *PHLDA2* was not expressed; from spleen, where *PHLDA2* was lowly expressed and not imprinted; and from allantois, where *PHLDA2* was highly expressed and imprinted (Figure S1). Interestingly, DNA upstream of *PHLDA2* was methylated at a higher level in allantois than in heart and spleen (Figure S1). In particular, the same CpG dinucleotide (CG1 in Figure 3) that was associated with *PHLDA2* expression in embryos was methylated at a higher level in allantois than in heart and spleen (Figure S1). A similar methylation pattern was also observed for another CpG site nearby (CG2; Figure S1).

### *PHLDA2* has Dosage-sensitive Effects on Bovine Pre-implantation Embryo

To test whether artificially suppressing expression of *PHLDA2* changes embryonic development, we injected fertilized zygotes with siRNA oligos targeting *PHLDA2* mRNA. Microinjection of 200 uM siRNA specific to *PHLDA2* resulted in a 10% development rate versus 26% for the control group (*P* = 0.0004), whereas microinjection of 150 uM siRNA resulted a development rate similar to the control group (Table 1). In contrast, microinjection of 100 uM siRNA caused an increase in blastocyst development (37%) relative to the control group (26%) (Table 1). To test the effect of the 100 uM siRNA injection on embryo development under different environmental conditions, oocytes were collected from ovaries in mid-summer during a period of heat stress where bovine embryo development shows a marked decrease. After fertilization, microinjection and subsequent culturing were performed. During the heat stress period, the development rate of blastocysts in the control group was 5% compared to 15% in the 100 uM-injected group (*P* = 0.02) (Table S1). In addition, there was a significant difference in cleavage rate for the 150 uM group (*P* = 0.047) compared to the control group. In a subsequent microinjection experiment done at the end of the heat stress season, the development rate of blastocysts was 25% in the 100 uM injected group compared to 20% and 18.35% in the control and sham groups, respectively (Table S2).

**Table 1:** Development rates for *PHILDA2* siRNA-injected and control embryo groups.

Treatment	Total	Cleaved	CleavageRate	Blastocysts	BlastocystRate
Control	279	208	74%^a^	54	26%^a^
Sham	249	191	77%^a^	49	26%^a^
100 uM	138	89	64%^a^	33	37%^a^
150 uM	65	45	69%^a^	11	24%^a^
200 uM	145	98	68%^a^	10	10%^b^

Differing superscripts within a column denote statistically significance difference at P<0.05.

### Knockdown of *CDKN1C* using siRNA Affects Bovine Pre-implantation Embryonic Development

**Figure 4:**
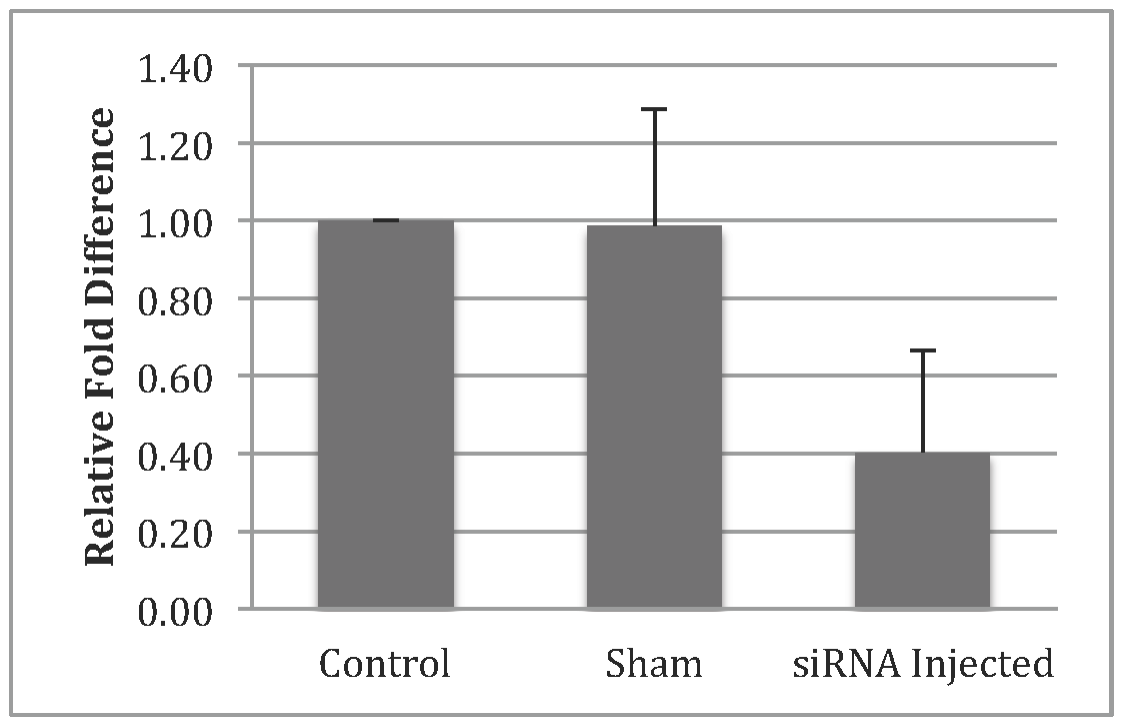
qRT-PCR results for control and *CDKN1C* siRNA injections. All samples were normalized to *GAPDH* using the ΔΔCt method [60].

We also tested the effect of *CDKN1C* knockdown on pre-implantation embryonic development. Initial siRNA experiments with different concentrations showed that 200 uM of siRNA produced ≥50% knockdown of *CDKN1C* gene expression in blastocysts collected on day 8 of culture. As such, this concentration was selected for further microinjection analysis. Injection of 200 uM *CDKN1C* siRNA resulted in 45% decrease in blastocyst rate (P<0.0006) by day 8 of development (Table 2). In contrast, injection of GFP specific siRNA did not result in a significant change in blastocyst rate (Table 2). In addition there was no significant difference in cleavage rate and blastocyst rate for the sham, siRNA injected, and control group in any of the IVF replications (P = 0.973). To determine the amount of knockdown achieved by siRNA injection, qRT-PCR was performed in the baseline control and the injected embryos. Figure 4 shows 50% knockdown in gene expression. Furthermore, phenotypic observations showed no marked difference between blastocysts produced from the baseline control or from *CDKN1C* injections (Figure S2).

**Table 2:** Development rates for *CDKN1C* siRNA-injected embryos and control embryo groups.

Treatment	Total^1^	Cleaved	Cleavage Rate	Blastocysts	Blastocyst Rate
Control	372	288	77%^a^	70	24%^a^
Sham	327	251	77%^a^	59	24%^a^
GFP control	225	148	67%^ b^	31	21%^ a^
*CDKN1C* siRNA 200 uM	354	272	77%^a^	34	13%^b^

1 Numbers represent average of six biological replicates.

Differing superscripts within a column denotes statistically significant differences (P<0.05).

### Transcriptomic Response of Embryos upon *CDKN1C* Knockdown

To better understand the mechanisms by which *CDKN1C* affects embryo survival, we analyzed the global RNA expression patterns in control, sham, and injected embryos using RNA-Seq. Comparative analysis of individual genes between sham- and siRNA-injected embryos revealed 51 genes that were differentially expressed between the two embryo groups (Table S3). GO analysis uncovered nine pathways significantly enriched for differentially expressed genes (FDR <0.10) between sham-injected and *CDKN1C* siRNA-injected embryos (Table 3). Of these, there appears to be a preponderance of perturbation to cellular signaling/communication (extracellular region, regulation of cell communication), nucleic acid processing (endonuclease activity, endoribonuclease activity, producing 3′-phosophomonoesters), and cellular metabolism (monosaccharide catabolic process, alcohol catabolic process, and various carbohydrate catabolic processes).

**Table 3:** Pathways significantly enriched (FDR <0.10) for differentially expressed genes between sham and *CDKN1C* siRNA-injected embryos.

GO ID	Ontology	Term
0005576	CC	Extracellular region
0016894	MF	Endonuclease activity, active with either ribo- or deoxyribonucleic acids and producing 3′-phosphomonoesters
0046365	BP	Monosaccharide catabolic process
0044421	CC	Extracellular region part
0010646	BP	Regulation of cell communication
0046164	BP	Alcohol catabolic process
0044275	BP	Cellular carbohydrate catabolic process
0016892	MF	endoribonuclease activity, producing 3′-phosphomonoesters
0016052	BP	Carbohydrate catabolic process

BP: Biological process; CC: Cellular component; MF: Molecular function Pathways are ranked in order of decreasing statistical significance.

## Discussion

### Association of DNA Methylation with Expression of *PHLDA2*

The regulatory mechanisms of *PHLDA2* expression and its imprinting are not well understood in cattle. DNA methylation is one of the most common mechanisms in regulating transcription. In mammals, a general rule is that methylation in promoter regions of genes represses transcription initiation whereas methylation in gene bodies enhances transcription elongation [20]. In bovine IVF embryos at the blastocyst stage, CpG sites within or near the *PHLDA2* gene were generally not methylated (Figure 3). Nevertheless, the methylation of a CpG site upstream of *PHLDA2* was associated with higher expression of the gene in degenerate embryos. This particular CpG site was near the boundary of the CpG island overlapping with *PHLDA2*. In fetal tissue DNA, *PHLDA2* was methylated at a higher level in heart, spleen, and allantois than in embryos, particularly in the region outside the CpG island. Interestingly, the same CpG site whose methylation correlated with expression in embryos also showed differential methylation between heart, spleen, and allantois DNA (Figure S1). While this CpG site was highly methylated in allantois, its methylation was lower in heart and spleen. *PHLDA2* expression in allantois was extremely high while it was relatively lower in spleen and not detected in heart. The differential methylation of the two CpG sites in these three tissues suggests correspondence with *PHLDA2* expression.

Recent studies have clearly established that while a negative correlation exists between DNA methylation in promoter regions and gene expression, intragenic methylation is abundant and that this methylation is positively correlated with gene expression [21]–[23]. Thus, the increase in gene expression observed when CG1 in degenerate embryos and CG1 and CG2 in allantois were methylated is consistent with these studies.

### Dosage Sensitivity of *PHLDA2* in Bovine Pre-implantation Embryos

Injections of 100 uM siRNA increased development from 26 to 37%, while more concentrated knockdown using 200 uM siRNA caused significant decrease in blastocyst rate (control 27% vs. 10%). This dosage-sensitive effect of *PHLDA2* was not only evident in our standard IVF system, but also in the presence of relatively adverse developmental conditions. Heat stress is a known condition that negatively affects the reproductive function in dairy cattle, which in turn leads to a decrease in embryonic development [24]–[27]. Production of IVF embryos during heat stress and in the end of heat stress season resulted in low blastocyst rates in the control group of non-injected embryos (Additional Files 2 and 3). In contrast, embryos that were injected with 100 uM *PHLDA2* siRNA had an increased blastocyst rate relative to controls. However, it should be acknowledged that the conditions under which embryos were produced were adverse. Interestingly, the 100uM siRNA group started out with a similar number of embryos to the 150uM group but produced significantly more embryos. Thus although small number of embryos was obtained under stress conditions, these were produced due to the inability for embryos to develop even with injection under the heat stress condition. However, these results (i.e., significant differences in blastocyst rate under heat stress) should be taken with caution due to the low number of blastocysts obtained and further pursued in future studies. Thus, regardless of environmental conditions, internal roles of *PHLDA2* can improve or adversely affect embryonic development during the pre-implantation period. As such, this not only reflects that *PHLDA2* has dosage sensitive functions, but that it also may be a key gene controlling early embryonic development in the bovine embryo.

Interestingly, *PHLDA2* has been suggested to serve as a developmental rheostat. In mice, overexpression of *Phlda2* has been shown to result in fetal and placental abnormalities while loss of *Phlda2* results in placentomegaly [28]. The results from this study reflect a similar pattern as increased expression of *PHLDA2* has been associated with early embryonic degeneration while reduced expression (200 uM siRNA) resulted in reduced blastocyst development. A slight reduction of *PHLDA2* expression appeared to rescue the effects of overexpression and restore development of the embryo suggesting that this gene indeed serves as a strong regulator of embryonic development. However, to provide further evidence of causality of embryo degeneration, further studies need to be done with biological replicates and with large sample size of injected embryos.

### Association of Expression Levels of Imprinted Genes with Embryo Development

Genes showing higher expression in degenerate embryos appear to have roles critical for cell division. *MKRN3* putatively functions as a ribonucleoprotein and been shown to be solely expressed after the maternal embryonic transition in bovine in-vitro embryos, where maternal stores of RNA are degraded and the embryonic transcripts have gained control [29], [30]. For *PEG3*, although its biological functions are still being determined, studies suggest a *Peg3/PAX-1* mediation of p53-mediated cell apoptosis [31]. Our results may be indicative of increased cell death and lack of RNA modification in the degenerate embryos and may give rise to suggested roles in the embryo’s developmental potential. Furthermore, *UBE3A* encodes for the E6-associated protein, which has a role as an ubiquitin ligase enzyme [32]. This is of interest as over-expression of this gene in degenerate embryos may result in an excess of polyubquitination and subsequent degradation of critical proteins by the proteosomes.

Of those genes found to have higher expression in blastocysts, reported functions are more limited. Studies suggest that *IGF2R* should be maintained at sufficient levels for proper development in utero [33]. In addition, low levels of *IGF2R* may result in developmental abnormalities such as large offspring syndrome [34]. *Tssc4* has been mapped to a region on chromosome 7 in the mouse that has association with early embryonic lethality (http://www.mousebook.org/index.php), but there is little known regarding its function in cattle.

Four genes (*USP29, NNAT, PEG10*, and *RTL1*) had very low expression in embryos making it unable to quantify differences accurately, while three genes (*IGF2, H19*, and *MIM1*) had undetectable levels of expression in our embryo populations. This may be due to expression levels being below detection limits or an absence of transcripts due to no expression of these genes during this developmental period. Although there is some evidence for expression of *H19* in mouse blastocysts [35], previous studies have shown that neither *H19* nor *IGF2* is expressed in bovine [5], [36] or ovine [37] blastocysts. One gene, *XIST*, was inconclusive in results due to sex bias as relative abundance for this gene has been shown to be higher in female blastocysts [38]. Thus, the use of pooling does not allow us to quantify this gene further.

An increasing number of studies have shown the importance and sensitivity of imprinted gene expression during the early developmental stages. A study by Tunster et al. [39] showed that a doubling of *Phlda2* expression in mice resulted in a 25–35% reduction in embryonic glycogen stores and a 13% decrease in embryonic weight. Furthermore, another study revealed that *Mash2-*deficient mouse embryos died due to placental failure by Day 10 postcoitum [40]. A prior study in mice revealed 26 imprinted genes aberrantly expressed in abnormally developed placentas [41]. Interestingly, all of these genes showed a 3-fold or less change in expression suggesting a heightened sensitivity of the organism to deviations from normal imprinted gene expression. As such, the relatively small fold changes found in this study could still hold biological importance when it comes to the functions of imprinted genes.

### Knockdown of *CDKN1C* Causes Reduced Blastocyst Development

*CDKN1C* (p57^KIP2^) is a member of the CIP/KIP family of cell-cycle inhibitors that have unique roles in embryogenesis [42]. Although the primary role of *CDKN1C* is cell cycle regulation, it has also been implicated to have roles that in apoptosis, cytoskeletal dynamics, and differentiation [43]. Mice lacking *Cdkn1c* have been found to have increased neonatal lethality and those surviving showed numerous developmental abnormalities ranging from cleft palates to abdominal defects [44]. Another study showed that most mice devoid of *Cdkn1c* expression died after birth, and tissues of these individuals had an increase in apoptotic cells and signs of delayed differentiation [45]. In contrast, excess of this gene has been shown to have association with increased embryonic lethality, suggesting dosage sensitivity during early developmental stages [46]. We hypothesized that by knocking down expression of *CDKN1C* using siRNA, embryonic development would be negatively affected, as the gene showed lower expression levels in degenerated embryos compared to blastocysts.

Knockdown of *CDKN1C* in the bovine pre-implantation embryo resulted in a 45% knockdown in blastocyst rate by day 8 of culture. Validation of knockdown was observed using qRT-PCR showing a 50% reduction in *CDKN1C* levels in day 8 blastocysts in comparison to sham and control groups. No significant effects were seen on cleavage rates. Given that *CDKN1C* knockdown resulted in reduced blastocyst formation suggests that this gene is a key factor driving early development. Specifically, these results imply that the differential expression that was observed between blastocyst and degenerates was causative, in part, to the embryos’ degeneration.

### Sensitivity of the Embryo to *CDKN1C* Dosage

Early embryonic development in mice has been shown to be extremely sensitive to the levels of *Cdkn1c* [46]. One excess copy caused growth retardation by E13.5 while loss of *Cdkn1c* resulted in overgrowth phenotypes [46]. Prior to differentiation into a blastocyst, individual blastomeres are totipotent; however, with differentiation the ICM cells gain pluripotentcy [47]. A study by Tury et al. [48] showed that *Cdkn1c* overexpression at E14.5–15.5 in mice resulted in a transition from proliferation to differentiation in neuronal tissues, whereas deficiency of this gene resulted in an opposite effect. Thus, a particular level of *CDKN1C* is required for proliferation to occur and then an increase in *CDKN1C* levels is required for a transition from cell division to differentiation. Indeed in our system there seems to be an apparent sensitivity to *CDKN1C* levels. Although cleavage rates were unaffected by knockdown, later development to the blastocyst stage was reduced significantly. This suggests that even low levels of *CDKN1C* were sufficient for early cell division but heightened levels may be needed for the transition to differentiation.

### Individual Genes and Pathways Perturbed by *CDKN1C* Knockdown

A total of 51 genes showed significant differential expression between sham- and siRNA-injected embryos. Notably, functions of the 20 differentially-expressed genes with the greatest differences (>10-fold expression difference between embryo groups) included 10 genes involved in apoptosis (*IFI6*, *IFI27*, *CCDC80*, *LUM*, *PHACTR3*, *GJA1*, *OLR1*, *CSRP3*, *NIM*, and *BMP2*). For example, *BMP2* can induce apoptosis by causing cell cycle arrest [49] and *LUM* may influence apoptosis through pathways regulated by Fas-Fas ligand interaction [50]. However, in addition to the functions of *IFI6* and *IFI27* in apoptosis, roles in viral infections have also been suggested [51]. Interestingly, the RNAi pathway in the cell can be induced by viral infection. Therefore, these two genes’ expression levels may be increased in the siRNA-injected group due to the *CDKN1C* siRNA introduction. All the apoptotic genes were highly expressed in injected compared to non-injected embryos probably as a result of the apoptotic activity in the injected embryos. Other functions of the genes found to be altered as a consequence of siRNA injection include lipid metabolism (*OLR1*, *FAT1*, *PLSCR1*, and *PARRES2*), differentiation (*CSRP3*, *CDH2*, and *BMP2*), and cell cycle regulation (*C27H8orf4*). Thus, knockdown of *CDKN1C* identified many genes that are regulated by this gene with a wide range of functions essential to the developing embryo.

Of the nine significant pathways found in the GO analysis, the endonuclease activity pathway was enriched with genes that showed higher expression in sham than in injected embryos. Decreased endonuclease activity has been shown to associate with increased DNA damage in neuronal development [52]. It has been shown that ablation of certain genes involved in DNA damage repair in murine embryos leads to embryonic lethality [53]. Decreased ribonuclease activity in *CDKN1C* knockdown embryos may be causing increased cellular damage and reduced development. Thus, p57 (the protein product for *CDKN1C*) may be a responder for DNA damage in the cell, and inadequate levels may prevent the embryo from recovering from potentially fatal replication mistakes. In addition, it appears that metabolic processes in the siRNA-injected embryos are altered in comparison to shams.

Seven out of eight differentially expressed genes (*ENO1, ENO3, GPI, PFKL, TPI1, DHDH, GALE*) for monosaccharide catabolic process were found to be significantly higher in sham embryos. Interestingly, *ENO1* and *ENO3* code for enolase, which is one of the last steps in glycolysis [54]. Glycolysis, which is a redox reaction, is a pathway that derives adenosine triphosphate (ATP), a main source of energy in the cell. It has been suggested that shifts in redox reactions can affect cell activities post-compaction and at differentiation, which are two critical processes in the bovine pre-implantation embryo [55]. In addition, enolase may serve as a receptor for ligands on the surface of the cell [56]. This is intriguing as the extracellular region pathway was also enriched for differentially expressed genes in this study. During the transition from morula to blastocyst, cells of the pre-implantation embryo undergo numerous changes including differentiation into specific cell types. Although there are numerous hypotheses regarding the mechanism of cell fate and differentiation during this time, cell signaling has been suggested as a key component [57]. Early studies have suggested that CDK inhibitors have functional roles in extracellular signaling and as such, reduction of *CDKN1C* may be altering factors on the cellular membrane responsible for cell signaling [58]. Thus, reducing the level of *CDKN1C* and preventing protein production via RNAi leads to global changes in the bovine pre-implantation embryo and uncovers new genes and pathways regulated by this gene beyond its immediate responsibilities in the cell cycle.

### Conclusions

This study revealed 10 differentially expressed imprinted genes between blastocyst and degenerate embryos, among which *PHLDA2* showed a higher expression level in degenerates than blastocysts and *CDKN1C* showed a higher expression level in blastocysts compared to degenerates. Knockdown of *PHLDA2* and *CDKN1C*–which are located in the same gene cluster–resulted in significant changes in embryo development using gene-specific siRNA injection into one-cell zygotes. RNA-Seq transcriptomic analysis of *CDKN1C*-siRNA injected vs. non-injected embryos revealed many genes and pathways that may be regulated by *CDKN1C* with functions critical to developing embryo. We conclude that *CDKN1C* and *PHLDA2* are major contributors to bovine embryo development.

## Methods

### In-vitro Fertilization, Embryo Culture, and Morphological Grading

Ovaries from cows were obtained from Applied Reproductive Technologies (Madison, WI) with a permission to use these ovaries for in vitro production of embryos, and upon arrival underwent aspiration of antral follicles (2–6 mm). Maturation of oocytes and fertilization were accomplished by combining sperm, heparin, and PHE with the oocytes as described in Khatib et al. [2]. Briefly, cumulus oocyte complexes were matured in M-199 supplemented with gonadotropins (FSH, LH, and estradiol), gentamicin, sodium pyruvate and 10% fetal bovine serum for 24 hours. After maturation, the cumulus oocyte complexes were washed in Tyrode's albumin lactate pyruvate(TALP)-Hepes buffer and transferred to fertilization media. Fertilization media consisted of IVF-TL (Specialty Media, Phillipsburg, NJ) supplemented with sodium pyruvate, gentamicin and fatty acid free bovine serum albumin(FAF-BSA). The oocytes were fertilized with frozen-thawed semen using the percol sperm separation technique described by [59] and adjusted to a final concentration of 1 million/ml. Once fertilized, the presumptive zygotes were incubated for 24 hours, then presumptive zygotes were stripped of their cumulus cells, washed once in TALP-Hepes. Those embryos designated as controls were directly placed in groups of 25 per 50 µL drop of SOF media (Specialty Media, Phillipsburg, NJ) supplemented with sodium pyruvate, gentamicin, FAF-BSA. Embryos designated as GFP control, sham or injected underwent the protocol described later for siRNA injection before being placed into SOF media for culture. At day 5 of development all embryos underwent morphological assessment via light microscopy, where in vitro-produced embryos should reach approximately 16–32 cells and show evidence of cellular compaction and coalescence by this time. Embryos failing to show both of these properties were deemed “early degenerates” and removed from the study. Culturing and incubation was then continued until day 8 of development when the second morphological evaluation was performed. Embryos with a fluid filled cavity (blastocoele) giving evidence of cellular differentiation into the ICM and trophectoderm, were classified as “blastocysts” (Figure S3). Embryos that showed compaction at day 5 but failed to form a blastocoele cavity were deemed as “late degenerates” (Figure S3). These two populations of embryos were used for analysis and collected into RNALater (Ambion, TX) solution to preserve RNA integrity. For initial transcriptomic analysis, four randomly sampled pools of embryos (two blastocyst and two degenerate pools) were created (n = 20 embryos/pool) using a single sire. A second set of pools (two blastocyst and two degenerate) was created (n = 20 embryos/pool) using a second sire.

Total RNA was extracted from the embryo pools using RNaqueous Micro (Ambion). Quality control of the RNA was performed using the RNA6000 NanoChip (Agilent Technologies, CA) with the Agilent 2100 Bioanalyzer to determine banding for 18S and 28S ribosomal RNA. Due to limitations in the amount of RNA in embryos, linear amplification was performed using the MessageAmp II aRNA amplification kit (Ambion). DNase I treatment was then performed using the RNaqueous Micro kit (Ambion) to ensure no genomic contamination in the samples.

### Quantitative Real-Time RT-PCR (qRT-PCR)

The cDNA was synthesized from the amplified RNA using the iScript cDNA synthesis kit (Bio-Rad Laboratories, CA). Primers for qRT-PCR were designed using Beacon Software (Premier Biosoft, CA) to span exons if possible (Table S4). Six genes had only one exon (*MAGEL2, MKRN3, NDN, H19, NAP1L5,* and *MIM1*), and as such, DNase treatment of the RNA pools prior to cDNA synthesis was used to ensure no genomic contamination. In addition, PCR reactions were performed with DNase-treated RNA samples using primers designed in introns of the gene 7-dehydrocholesterol reductase (*DHCR7*) to test for the presence of genomic DNA in RNA samples. After two rounds of DNase treatment, no product was observed deeming the samples free of DNA contamination. An internal control gene for normalization was chosen using the Vandesompele method [60]. Briefly, three genes (Beta actin- *B-ACTIN;* ribosomal protein, large, P0, RPLP0; and glyceraldehyde phosphate dehydrogenase-*GAPDH*) were tested for stability across all samples. The gene with the lowest stability value (*GAPDH*, M = 0.26) was chosen as an adequate housekeeping gene for further assays. All imprinted genes underwent initial expression analysis to determine transcript abundance and those showing expression were then subsequently tested between morphological groups to quantify differential expression utilizing the ECO real-time PCR system (Illumina, San Diego, CA). The relative gene expression values were calculated using the 2^−ΔΔCt^ method [61]. Statistical analysis was performed using R version 2.15.2 (www.r-project.org/). The expression analysis for differentially expressed imprinted genes was analyzed using analysis of variance (ANOVA) of ΔCt values to determine possible sire and morphological effects.

### Analysis of DNA Methylation of *PHLDA2* by Bisulfite Sequencing

To evaluate the methylation status of *PHLDA2*, genomic DNA from embryo pools and tissues was treated with bisulfite and purified using the Epitect Bisulfite Kit (Qiagen). We performed the methylation analysis on pools of 20 blastocysts or degenerates. One pool for each of the developmental statuses was evaluated. Nonetheless, embryos were collected at multiple times to obtain the sufficient number of embryos in the pools. Bisulfite-treated DNA was first amplified by a PCR reaction for 40 cycles. The PCR products were gel purified and amplified in a second PCR reaction for 18 cycles using the same primers. The PCR products were gel purified, ligated to the pGEM-T Vector (Promega, WI), and transformed into JM101 competent cells (Promega) following the manufacturer’s instructions. Bacterial colonies were screened for the presence of a single copy of insert fragment by PCR with primers pairing with vector sequences flanking the TA cloning site. Each embryo on average contained 100–150 cells. Thus there were a total of approximately 4000–6000 chromosomes in the pool. We sequenced ∼20 clones and selected only those with the highest quality of sequence before looked at their actual sequences. The PCR products were sequenced to obtain bisulfite-converted DNA sequences. Association between DNA methylation at each CpG site with developmental status or tissue was tested by Fisher’s exact test. Only CpG sites with a combined (two samples in comparison) methylation level between 10% and 90% were tested, and statistical significance was declared at a P<0.01.

### siRNA Design and Synthesis

siRNA sequences were designed and synthesized by Sigma-Aldrich (St. Louis, MO). A BLAST of siRNA sequences was done against the bovine mRNA reference sequence to ensure that there would be minimal off-target effects. Research has shown that naturally produced siRNA have a 5′-phosphate on the antisense strand that participates in activation of the RNA induced silencing complex (RISC) [62]. Although most synthetically-produced siRNAs have a 5′-OH (antisense), ATP-dependent phosphorylation occurs shortly after introduction into the cell [62]. Given that we are injecting siRNA into a newly developing embryo with limited stores of ATP, a 5′-Phosphorylation modification was added to minimize reduction of energy stores in the embryo. The siRNA duplex sequences were *PHLDA2* antisense [Phos]AGUAGCACCGGGCUAUAUCdTdT, *PHLDA2* Sense GAUAUAGCCCGGUGCUACUdTdT, *CDKN1C* antisense [Phos]AAAUCCCUGAGUGCGGCGGdTdT, and *CDKN1C* sense CCGCCGCACUCAGGGAUUUdTdT. For siRNA injection three concentrations were assessed (100 uM, 150 uM, 200 uM).

### Experimental Controls

Two control groups were designed to help infer effects of single-gene knockdown. In the first control group (sham control), embryos underwent mechanical puncturing by the microinjection needle but no siRNA delivery in order to assure that potential phenotypic observations are due to the siRNA, and not a result of mechanical damage due to injection. The second group was a baseline control that consisted of embryos produced by our conventional IVF system. The third control group consisted of embryos that were injected with an siRNA specific to green fluorescent protein (GFP). A sequence that is not native to mammalian species, GFP serves as a control to ensure that any effects due to siRNA knockdown are gene specific. The sequences used for GFP siRNA are as follows: GFP-sense GCCACAACGUCUAUAUCAUdTdT and GFP-Antisense [Phos]AUGAUAUAGACGUUGUGGCdTdT. A concentration of 200 uM GFP siRNA was chosen for subsequent analysis and delivered in the same volume (∼7–10 picoliters) as the remaining siRNA.

### Embryo Preparation and Microinjection of siRNA

In vitro maturation followed the protocol outlined in Khatib et al. [2] except that putative zygotes were incubated for 18-hours. After this period, zygotes were removed from fertilization media, denuded of cumulus-complexes, and then prepared for microinjection. Microinjection of the zygotes was performed using an inverted Nikon Diaphot microscope (200x magnification) as described by Nganvongpanit et al. [15] with some modifications. In summary, a group of 25–35 zygotes were placed in a droplet of TALP-Hepes wash media with a mineral oil overlay. A microinjection needle was used to pierce the zona pellucida and deposit approximately ∼7–10 picoliters of siRNA into the cytoplasm of the zygote using the MINJ-D pressurized microinjection system (Tritech Research, CA). Zygotes were then washed twice in TALP-HEPES wash media post-injection and placed in SOF culture medium and incubated under the parameters mentioned above.

### Morphological Grading

Cleavage rates were assessed as a marker of fertilization for this study. In addition, embryos were assessed on day 8 of culture for blastocyst development. Embryos at the blastocyst stage were collected from each treatment group and underwent RNA extraction, cDNA synthesis, and qRT-PCR as outlined in the previous sections. Comparison of cleavage and blastocyst rates was completed using a chi-squared test for contingency with statistical significance deemed at P<0.05.

### RNA-Seq Pathway Analysis of siRNA and Non-siRNA Injected Embryos

Total RNA from control, sham-injected, and siRNA-injected *CDKN1C* embryo pools underwent amplification using the MessageAmp II aRNA Amplification Kit (Ambion). Libraries of amplified RNA for each pool were prepared following the Illumina mRNA-Seq protocol. Sequencing libraries were created from 50 ng samples and sequenced with Illumina’s HiSeq 2000 at the University of Wisconsin-Madison Biotechnology Center. Mapping reads to the bovine reference genome, assembly of transcripts and estimation of differential expression, and Gene Ontology (GO) enrichment analysis was performed as described in Driver et al. [63]. Sequencing reads were mapped to the reference genome (bosTau7) using the software package Tophat (v2.0.4) [64]. Cufflinks (v2.0.2) was used to assemble transcript models from alignments and to estimate their abundance in the transcriptome [65]. Abundances of transcripts were upper-quartile normalized and also corrected for sequence bias in order to improve expression estimates [66]. Differential expression of genes was tested using Cuffdiff, a tool part of the Cufflinks package for testing differential gene expression [64]. In addition, GO enrichment analysis was performed using the GOseq (v1.8.0) package [67] that is available in the R language/environment. Biological pathways with a FDR <0.10 were considered significant.

## Supporting Information

Figure S1 **DNA methylation of PHLDA2 in bovine tissues.** Clones from allantois, heart, and spleen DNA are shown in red, black, and blue, respectively.
(TIFF)

Figure S2 **Morphological comparison of blastocysts on day 8 of culture.** Box A (left) shows a representative sample of blastocysts that were injected with CDKN1C siRNA. Box B (right) shows representative samples of control (non-injected) blastocysts.
(TIFF)

Figure S3 **Morphological assessment of embryos.** Compacted morulas (A) that were cultured until day 8 of development and either showed signs of blastocoele formation (B) or degeneration (C).
(TIFF)

Table S1 **Injection of 100 uM and 150 uM PHLDA2 siRNA under heat stress.**
(DOC)

Table S2 **Injection of 100 uM and 150 uM** ***PHLDA2*** **siRNA under presumptive post-heat stress.**
(DOC)

Table S3 **Genes that were differentially expressed between sham and injected embryos using RNA-seq.**
(DOC)

Table S4 **Primer sequences for real-time PCR reactions and product sizes.**
(DOC)
