# Supplementary figures and images for: Knockdown of CDKN1C (p57kip2) and PHLDA2 Results in Developmental Changes in Bovine Pre-implantation Embryos
Source: PLoS One. 2013 Jul 22;8(7):e69490. doi: 10.1371/journal.pone.0069490 (PMC3718760; doi:10.1371/journal.pone.0069490)

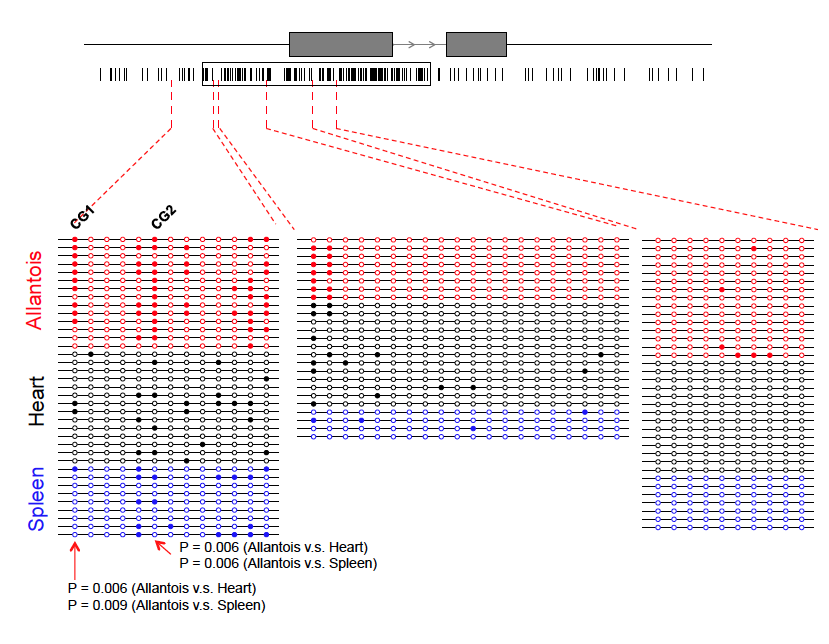

Supplement: Figure S1 — DNA methylation of PHLDA2 in bovine tissues. Clones from allantois, heart, and spleen DNA are shown in red, black, and blue, respectively. (TIFF) [file pone.0069490.s001.tiff]

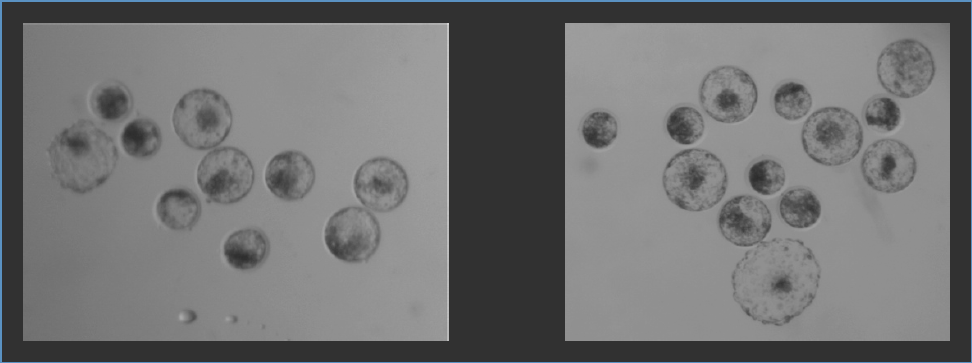

Supplement: Figure S2 — Morphological comparison of blastocysts on day 8 of culture. Box A (left) shows a representative sample of blastocysts that were injected with CDKN1C siRNA. Box B (right) shows representative samples of control (non-injected) blastocysts. (TIFF) [file pone.0069490.s002.tiff]

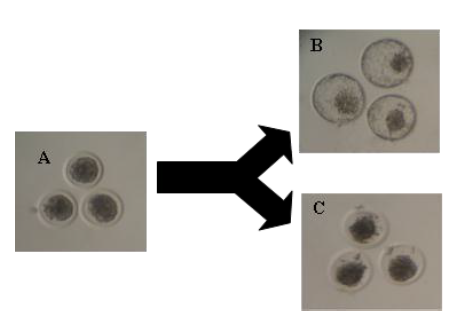

Supplement: Figure S3 — Morphological assessment of embryos. Compacted morulas (A) that were cultured until day 8 of development and either showed signs of blastocoele formation (B) or degeneration (C). (TIFF) [file pone.0069490.s003.tiff]
