# Supplementary material for: Knockdown of CDKN1C (p57kip2) and PHLDA2 Results in Developmental Changes in Bovine Pre-implantation Embryos
Source: PLoS One. 2013 Jul 22;8(7):e69490. doi: 10.1371/journal.pone.0069490 (PMC3718760; doi:10.1371/journal.pone.0069490)
Supplement: Table S1 — Injection of 100 uM and 150 uM PHLDA2 siRNA under heat stress. (DOC) [file pone.0069490.s004.doc]

**Table S1. Injection of 100 uM and 150** uM PHLDA2 siRNA under heat stress

| Treatment Group | Total | Cleaved | Cleavage Rate | Blastocysts | Blastocyst Rate |
| --- | --- | --- | --- | --- | --- |
| Control | 166 | 123 | 68%^a^ | 6 | 5%^a^ |
| *PHLDA2* siRNA 100 uM | 70 | 48 | 68%^a^ | 8 | 15%^b^ |
| *PHLDA2* siRNA 150 uM | 74 | 43 | 61%^b^ | 2 | 4%^a^ |

Differing superscripts within a column denote statistically significant differences (*P* < 0.05).
