# Supplementary material for: Knockdown of CDKN1C (p57kip2) and PHLDA2 Results in Developmental Changes in Bovine Pre-implantation Embryos
Source: PLoS One. 2013 Jul 22;8(7):e69490. doi: 10.1371/journal.pone.0069490 (PMC3718760; doi:10.1371/journal.pone.0069490)
Supplement: Table S2 — Injection of 100 uM and 150 uM PHLDA2 siRNA under presumptive post-heat stress. (DOC) [file pone.0069490.s005.doc]

**Table S2. Injection of 100 uM and 150 uM *PHLDA2*** siRNA under presumptive post-heat stress

| Treatment Group | Total | Cleaved | Cleavage Rate | Blastocysts | Blastocyst Rate |
| --- | --- | --- | --- | --- | --- |
| Control  Sham | 105  96 | 70  54 | 66.7%  56.25% | 14  10 | 20%  18.25% |
| *PHLDA2* siRNA 100 uM | 97 | 64 | 65.98% | 16 | 25% |
| *PHLDA2* siRNA 150 uM | 125 | 91 | 72.8% | 17 | 18.68% |
