# Supplementary material for: Knockdown of CDKN1C (p57kip2) and PHLDA2 Results in Developmental Changes in Bovine Pre-implantation Embryos
Source: PLoS One. 2013 Jul 22;8(7):e69490. doi: 10.1371/journal.pone.0069490 (PMC3718760; doi:10.1371/journal.pone.0069490)
Supplement: Table S3 — Genes that were differentially expressed between sham and injected embryos using RNA-seq. (DOC) [file pone.0069490.s006.doc]

| Gene | Locus(bosTau7) | log2(fold_change) | fold_change | Overexpression | qvalue | qvalue |
| --- | --- | --- | --- | --- | --- | --- |
| IFI6 | chr2:131115858-131119447 | -6.32041 | 79.92 | I | 2.04E-07 | 0.000 |
| IFI27 | chr21:59014080-59022973 | -6.16491 | 71.75 | I | 0.0285423 | 0.029 |
| BID | chr5:115412156-115418905 | 5.43679 | 43.31 | S | 0.00611461 | 0.006 |
| CCDC80 | chr1:58080017-58115541 | -5.26498 | 38.45 | I | 0.00536579 | 0.005 |
| ISG15 | chr16:48676752-48677780 | -5.20855 | 36.98 | I | 0.0123406 | 0.012 |
| LUM | chr5:23650318-23657534 | -5.10874 | 34.51 | I | 6.68E-05 | 0.000 |
| PHACTR3 | chr13:57213274-57279363 | -5.00485 | 32.11 | I | 0.104192 | 0.104 |
| LOC100335809 | chr2:6670518-6681740 | -4.95429 | 31.00 | I | 7.00E-05 | 0.000 |
| GJA1 | chr9:31507202-31520216 | -4.88782 | 29.61 | I | 0.000315737 | 0.000 |
| XCL1 | chr16:33520304-33523470 | -4.82608 | 28.37 | I | 0.00112997 | 0.001 |
| C27H8orf4 | chr27:37380049-37381351 | -4.55854 | 23.56 | I | 0.000833725 | 0.001 |
| AMY2B | chr3:42379420-42402070 | -4.54654 | 23.37 | I | 0.0519033 | 0.052 |
| LOC510631 | chr7:41563878-41567475 | -4.40798 | 21.23 | I | 6.68E-05 | 0.000 |
| RARRES2 | chr4:116419569-116422625 | -4.3782 | 20.80 | I | 0.000833725 | 0.001 |
| PLOD2 | chr1:124617975-124739559 | -3.91289 | 15.06 | I | 0.000151231 | 0.000 |
| OLR1 | chr5:106703848-106715154 | -3.7881 | 13.81 | I | 0.0667877 | 0.067 |
| CSRP3 | chr29:26759118-26779797 | -3.69731 | 12.97 | I | 0.104192 | 0.104 |
| VIM | chr13:31432087-31440017 | -3.66348 | 12.67 | I | 0.00611461 | 0.006 |
| CDH2 | chr24:29398109-29646615 | -3.55893 | 11.79 | I | 0.00611461 | 0.006 |
| BMP2 | chr13:49207397-49218698 | -3.36487 | 10.30 | I | 0.0857017 | 0.086 |
| GATM | chr10:66442207-66458222 | -3.25909 | 9.57 | I | 0.0015428 | 0.002 |
| TST | chr5:80585175-80591756 | 3.19801 | 9.18 | S | 0.172886 | 0.173 |
| RNASEH2A | chr7:10989463-10996393 | 3.17779 | 9.05 | S | 0.104192 | 0.104 |
| LOC515823 | chr10:75128125-75129562 | 3.1723 | 9.01 | S | 0.11784 | 0.118 |
| ETHE1 | chr18:51364563-51383878 | 3.12143 | 8.70 | S | 0.0133299 | 0.013 |
| LOC100850219 | chr5:25327382-25336652 | -2.94068 | 7.68 | I | 0.0159411 | 0.016 |
| LOC788610 | chr15:47867002-47868681 | 2.90533 | 7.49 | S | 0.0667877 | 0.067 |
| HBG | chr15:47852891-47854506 | 2.88532 | 7.39 | S | 0.0527532 | 0.053 |
| - | chrUn_AAFC03100583:48768-54903 | 2.85006 | 7.21 | S | 0.194133 | 0.194 |
| LOC100849362 | chrY:3503924-3652372 | 2.83774 | 7.15 | S | 0.0298012 | 0.030 |
| SLC7A4 | chr17:75507216-75510133 | 2.81633 | 7.04 | S | 0.129536 | 0.130 |
| UCHL1 | chr6:62454747-62466453 | -2.79919 | 6.96 | I | 0.162185 | 0.162 |
| SLITRK2 | chrX:17410200-17413598 | -2.74863 | 6.72 | I | 0.15962 | 0.160 |
| LOC507211 | chr9:31659066-31826452 | 2.71243 | 6.55 | S | 0.11784 | 0.118 |
| GLCCI1 | chr4:16107848-16217411 | -2.70933 | 6.54 | I | 0.194133 | 0.194 |
| BIRC3 | chr15:5391794-5422552 | -2.70615 | 6.53 | I | 0.0667877 | 0.067 |
| PLSCR1 | chr1:124197036-124224862 | -2.67092 | 6.37 | I | 0.0519033 | 0.052 |
| FAM110A | chr13:60912609-60926339 | 2.66448 | 6.34 | S | 0.129536 | 0.130 |
| WBP1 | chr11:10572506-10574898 | 2.656 | 6.30 | S | 0.104192 | 0.104 |
| KIF7 | chr21:20902079-20919879 | -2.54852 | 5.85 | I | 0.0386798 | 0.039 |
| SRM | chr16:39298009-39302069 | 2.40596 | 5.30 | S | 0.0800995 | 0.080 |
| XIST | chrX:47265732-47302267 | -2.34036 | 5.06 | I | 0.0671875 | 0.067 |
| CIB1 | chr21:21450599-21453772 | 2.32354 | 5.01 | S | 0.0826982 | 0.083 |
| TPRG1L | chr16:46723485-46726372 | 2.30358 | 4.94 | S | 0.118303 | 0.118 |
| RNASE1 | chr10:25672191-25673775 | 2.29757 | 4.92 | S | 0.171802 | 0.172 |
| CCNL1 | chr1:111988773-112002656 | -2.2819 | 4.86 | I | 0.11784 | 0.118 |
| NID2 | chr10:44859533-44958463 | -2.23342 | 4.70 | I | 0.121965 | 0.122 |
| AP2S1 | chr18:53642812-53652534 | 2.23307 | 4.70 | S | 0.171802 | 0.172 |
| OGT | chrX:48728453-48754810 | -2.13832 | 4.40 | I | 0.195152 | 0.195 |
| FAT1 | chr27:17621364-17747563 | -2.1212 | 4.35 | I | 0.15962 | 0.160 |
| IFNT2 | chr8:23602560-23603873 | -2.11584 | 4.33 | I | 0.15962 | 0.160 |
|  |  |  |  | S- SHAM |  |  |
|  |  |  |  | I- INJECTED |  |  |
