# Supplementary material for: Knockdown of CDKN1C (p57kip2) and PHLDA2 Results in Developmental Changes in Bovine Pre-implantation Embryos
Source: PLoS One. 2013 Jul 22;8(7):e69490. doi: 10.1371/journal.pone.0069490 (PMC3718760; doi:10.1371/journal.pone.0069490)
Supplement: Table S4 — Primer sequences for real-time PCR reactions and product sizes. (DOC) [file pone.0069490.s007.doc]

**Table S4. Primer sequences for real-time PCR reactions and product sizes.**

| Gene | Primer | Sequence (5’-3’) | Amplicon (bp) |
| --- | --- | --- | --- |
| *UBE3A* | Forward | GGGACTCTGTTGTGATTAGGG | 171 |
|  | Reverse | TAGGTAACCTTTCTGTGTCTGG |  |
| *NDN^a^* | Forward | AACGTGCTGCGCATCTTG | 103 |
|  | Reverse | TCAGGTAGTTCTGCTGGACGAA |  |
| *MAGEL2^b^* | Forward | CTGATGGTGGTTCTGAGCCT | 257 |
|  | Reverse | CAGGACAATCATCTTGCTGG |  |
| *MKRN3* | Forward | CTGCAGACAGCGGCCCTAGC | 222 |
|  | Reverse | CCCGGTAGGGTTGCCCAGGA |  |
| *CDKN1C* | Forward | CAAGCGGCTGCGATGAGAG | 67 |
|  | Reverse | TCCTGTCCACTGCCCAACG |  |
| *IGF2R* | Forward | CAGTCGCAAAGTCGGAACC | 140 |
|  | Reverse | GGTCACAGTGGAAGAAGATGG |  |
| *PEG3^c^* | Forward | CGCCAAAGTCAGGGAGAG | 150 |
|  | Reverse | CTTAACTGCCAGGACACC |  |
| *NAP1L5* | Forward | TCCTTTCGTCACAGTATCGC | 118 |
|  | Reverse | TGAGTTCTGCTGCTGCTG |  |
| *TSSC4* | Forward | TGCCACCAAGAACCTTCG | 106 |
|  | Reverse | CCTCTGCCATGTGTCACC |  |
| *PEG10* | Forward | CTTTCCAGCCTTCGCAGAG | 126 |
|  | Reverse | CTTCACTCCTGTGGCAATGG |  |
| *USP29* | Forward | AGGAGGAAGTTCCCTTTGTTGC | 143 |
|  | Reverse | TCTCTGTGACGGCTGAAATAGC |  |
| *RTL1* | Forward | CCCTCCTCTACCACCCCAAG | 107 |
|  | Reverse | CTTGCCCGTCCGCTTGTC |  |
| *NNAT* | Forward | CACCCACCCACCAGTCTC | 142 |
|  | Reverse | TTCTCGACACCGTGTATGC |  |
| *MIM1* | Forward | ACTCGGTTGTCAGTCACAC | 115 |
|  | Reverse | GAATTTCCATCGTCTTATTAGC |  |
| *IGF2* | Forward | TGCTGCTATGCTGCTTACC | 151 |
|  | Reverse | AACACTCTTCCACGATGCC |  |
| *H19* | Forward | CGTTCCTTTAGTCTCCTGAC | 119 |
|  | Reverse | AGTCCGTGTTCCAAGTCC |  |
| *XIST* | Forward | CCACTGAGCAACAACTCTAGG | 126 |
|  | Reverse | GGCAAATATGAAGGGAACAACC |  |
| *RPLP0* | Forward | GACAATGGCAGCATCTAC | 198 |
|  | Reverse | GAAGGTGTAATCAGTCTCC |  |
| *B-ACTIN* | Forward | AGGCCAACCGTGAGAAGATGAC | 100 |
|  | Reverse | CCAGAGGCATACAGGGACAGC |  |
| *GAPDH* | Forward | TGCCCAGAATATCATCCC | 134 |
|  | Reverse | AGGTCAGATCCACAACAG |  |

^a^*NDN* primers referenced from Wee et al. [68]

^b^*MAGEL2* primers referenced from Tveden-Nyborg et al. [30]

^c^*PEG3* primers referenced from Katz-Jaffee et al. (2008)
